# Costs and Effectiveness of Treatment Alternatives for Proximal Caries Lesions

**DOI:** 10.1371/journal.pone.0086992

**Published:** 2014-01-27

**Authors:** Falk Schwendicke, Hendrik Meyer-Lueckel, Michael Stolpe, Christof Edmund Dörfer, Sebastian Paris

**Affiliations:** 1 Department of Operative and Preventive Dentistry, Charité - Universitätsmedizin Berlin, Berlin, Germany; 2 Department of Operative Dentistry, Periodontology and Preventive Dentistry, RWTH Aachen University, Aachen, Germany; 3 Kiel Institute for the World Economy, Kiel, Germany; 4 Clinic for Conservative Dentistry and Periodontology, Christian-Albrechts-University, Kiel, Germany; Aix Marseille Université, France

## Abstract

**Objectives:**

Invasive therapy of proximal caries lesions initiates a cascade of re-treatment cycles with increasing loss of dental hard tissue. Non- and micro-invasive treatment aim at delaying this cascade and may thus reduce both the health and economic burden of such lesions. This study compared the costs and effectiveness of alternative treatments of proximal caries lesions.

**Methods:**

A Markov-process model was used to simulate the events following the treatment of a proximal posterior lesion (E2/D1) in a 20-year-old patient in Germany. We compared three interventions (non-invasive; micro-invasive using resin infiltration; invasive using composite restoration). We calculated the risk of complications of initial and possible follow-up treatments and modelled time-dependent non-linear transition probabilities. Costs were calculated based on item-fee catalogues in Germany. Monte-Carlo-microsimulations were performed to compare cost-effectiveness of non- versus micro-invasive treatment and to analyse lifetime costs of all three treatments.

**Results:**

Micro-invasive treatment was both more costly and more effective than non-invasive therapy, with ceiling-value-thresholds for willingness-to-pay between 16.73 € for E2 and 1.57 € for D1 lesions. Invasive treatment was the most costly strategy. Calculated costs and effectiveness were sensitive to lesion stage, patient’s age, discounting rate and assumed initial treatment costs.

**Conclusions:**

Non- and micro-invasive treatments have lower long-term costs than invasive therapy of proximal lesions. Micro-invasive therapy had the highest cost-effectiveness for treating D1 lesions in young patients. Decision makers with a willingness-to-pay over 16.73 € and 1.57 € for E2 and D1 lesions, respectively, will find micro-invasive treatment more cost-effective than non-invasive therapy.

## Introduction

With the prevalence of cavitated proximal caries lesions declining in most industrialised countries [1], [2], the majority of proximal lesions are non-cavitated enamel- or enamel-dentin lesions [3], [4]. The prevalence of such lesions has been reported to be 39% at the age of 12, increasing to 72% at the age of 20–21 [5], with most of these lesions being active and slowly but continuously progressing [4], [5]. The distribution of such lesions is highly skewed, with a minority of patients showing the majority of lesions [3]. Until recently, the treatment of non-cavitated proximal lesions was performed either non-invasively using oral hygiene education measures or repeated fluoride application, or invasively including caries removal and restoration of the cavity. Whilst the first approach preserves dental substance, it is highly dependent on patient’s compliance. The second approach, in contrast, does not greatly depend on the patient’s cooperation, but involves substantial loss of dental tissue, especially if proximal lesions are restored, and is usually the start of a cycle of re-interventions due to the limited longevity of dental restorations [6]. This vicious cycle of re-restorations is associated with a further and increasing loss of tooth substance and can compromise both the vitality and retention of the treated tooth [7].

Resin infiltration is a micro-invasive option to treat non-cavitated proximal lesions. It exploits capillary forces to transport resins with high penetration coefficients into enamel porosities. After polymerisation, the infiltrant occludes diffusion pathways for cariogenic acids and dissolved minerals [8]. Such micro-invasive treatment of enamel caries has been clinically proven efficacious to arrest and stabilize lesions [9], [10]. Thus, resin infiltration may be suitable to prevent or delay the described cycle of re-restorations, eventually retaining teeth for longer and reducing long-term costs compared with alternative therapies. The present study analysed how successful non- and micro-invasive treatments are in delaying the restoration of the tooth, and which initial and follow-up costs accrue over the lifetime of the patient. Additionally, we compared the long-term costs of all three management strategies (non-invasive, micro-invasive, invasive treatment) emanating from both the initial and possible re-treatments.

## Materials and Methods

### Model

We simulated the treatment of proximal posterior lesions with radiographic extension into the enamel (E2) or outer third of the dentin (D1) [11] in permanent teeth. We compared three interventions within the context of German healthcare:

Non-invasive treatment, including for example oral hygiene education, flossing advice or topical fluoridation
Micro-invasive treatment using resin infiltration
Invasive treatment using occlusal-approximal composite restoration

We constructed a Markov model for each intervention (TreeAge Pro 2013, TreeAge Software, Williamstown, MA, USA), consisting of the initial and follow-up health states. The likelihood of lesions translating to the next health state was based on transition probabilities. Each translation was performed by traversing treatment states, thereby accruing costs.

**Figure 1:**
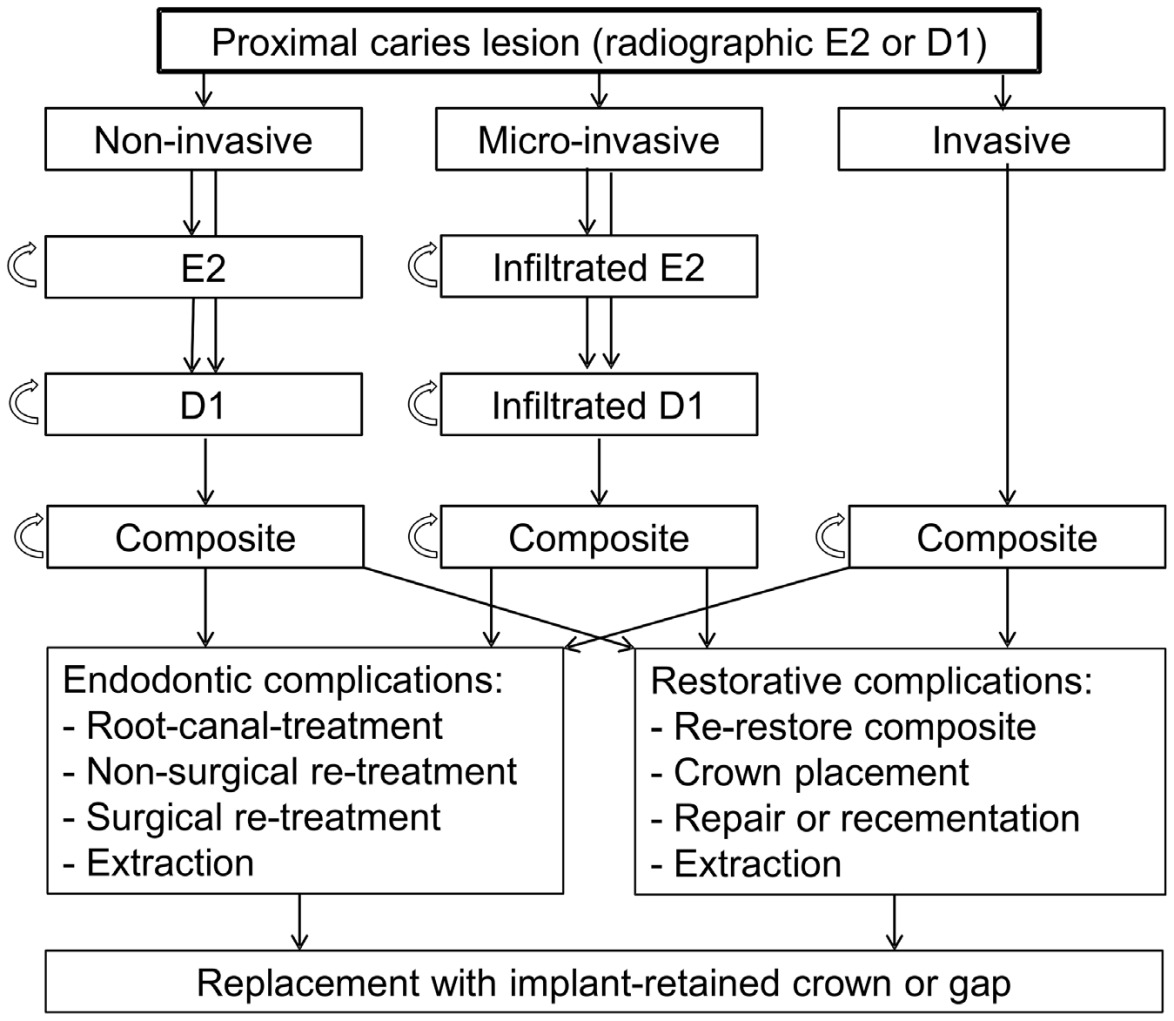
State-transition diagram. A Markov-model was used to simulate non-, micro- or invasive treatment of proximal E2 or D1 lesions. Non- and micro-invasively treated E2 lesions remained in their state (circled arrows) or progressed to D1 lesions according to their transition probabilities (Table 1). Translation to the next state accrued costs (Table 2). If D1 lesions progressed further, restoration with composite was simulated. Invasively treated lesions were restored using composite regardless of their stage. Restorations were assumed to fail either due to endodontic complications, requiring endodontic (re-)treatment, or due to restorative complications, requiring repair, recementation or re-restoration. Teeth could always translate to extraction (depending on allocation probabilities or if no further options remained). Missing teeth were replaced in 80% of simulations. Replacement was performed using implant-retained single crowns.

Non- or micro-invasively treated lesions were assumed to progress stepwise to the next radiographic stage (E2→D1→D2), with lesions extending beyond the outer third of the dentin being restored. Lesions treated invasively were immediately restored at the beginning of the simulation. Simulation was performed in 6-monthly cycles, with the sequence of events constructed according to current evidence and the consultation of an expert panel at Charité - Universitätsmedizin Berlin, RWTH Aachen and CAU Kiel (FS, HML, CD, SP). We modelled only complications related to the treatment of proximal caries lesions and did not, for example, simulate periodontal complications (Fig. 1). Model validation was performed internally (by varying distributions and key parameters to check their impact on the results) and externally (peer reviewing by an experienced health economist [MS]).

### Estimation of Parameters

We estimated transition probabilities of non-invasively treated lesions based on caries risks reported by a long-term cohort study from Sweden [12]. Patients in this study received tooth brushing instructions, repeated fluoride application, dietary counselling, or fluoride lozenges or gums depending on their caries risk and the time period within the study [13]. The progression probability of infiltrated lesions was calculated based on published clinical trials investigating the efficacy of resin infiltration in permanent teeth [14], [15]. Risk of bias was assessed to estimate the evidence supporting our simulation. We calculated Risk Ratios (RR) and 95% Confidence Intervals (95% CI) as effect estimates to compare the risk of progression in infiltrated and non-invasively treated lesions. Since lesion progression was assumed to depend on the patient’s age, we used age-dependent hazard functions to determine transition probabilities of both non-invasively and micro-invasively treated lesions. Hazard functions were calculated by non-linear least-square regression of transition probabilities at different ages following the function p = n×αk, with α denoting the patient’s age and p the transition probability per cycle (SPSS 20, IBM, Chicago, USA).

Risk of failure of composite restorations and all subsequent health states was estimated from a non-systematic literature review. Mean annual failure rates (AFR) and 95% CI were calculated as effect estimates. Since risk of failure was assumed to vary according to the time spent in each health state, we extracted AFRs for three different time plateaus (0–2, 2–5, >5 years spent in the health state) and re-calculated them into transition probabilities per 6-month cycle using the formula p = 1−(1−ā××y)^(1/(2y))^, with p denoting transition probability per cycle, ā the mean annual failure rate for the respective time period and y the time-plateau in years (*e.g.* 2 for 0–2 years). Allocation probabilities (i.e. allocating a tooth to a certain treatment after complications) were estimated from reviewed studies, with final consensus obtained by the panel.

The model adopted a mixed public-private-payer perspective, as characteristic in German healthcare. Cost calculations were mainly based on the Public and Private Dental Fee Catalogues, BEMA and GOZ [16]. BEMA defines fee items within the public insurance, which covers 88% of insured Germans [17], with only few treatments not being covered or reimbursed (resin infiltration, posterior composites, non-surgical re-root-canal-treatment, implant-retained crowns). For these items, calculation was based on GOZ. Since factoring of chargeable item-points is common to determine prices of private treatment in Germany, the standard multiplication factor (×2.3) was used within base-case-analysis. Items were restricted in number and character to reflect cost limitations and awareness. Total costs per course of treatment were calculated based on the quantification (q) of itemised costs (c): c_1_×q_1_+ c_2_×q_2_ etc. Costs were calculated in Euro (€) and future costs discounted at 3% per annum [18]. No such discounting was performed for future effectiveness.

### Cost- and Effectiveness-analyses

To analyse costs and effectiveness of different treatments, we performed two analyses: The first analysis was a cost-effectiveness-analysis, comparatively evaluating how successful non- and micro-invasive treatments are in delaying the restoration of the tooth, i.e. how many proximal lesions remain unrestored at which costs over the lifetime of a patient. Secondly, we performed a cost-analysis comparing the life-long costs of the initial- and re-treatment of a proximal lesion using different strategies. Both analyses investigated E2 and D1 lesions separately. Within the base-case scenario, we simulated the treatment of one proximal lesion in a 20-year-old male German patient with a remaining life expectancy of 58.25 years [19].

Initial examinations did not generate any costs, since they were performed before the treatment itself and thus independent from the chosen strategy. Non-invasive treatment generated costs for topical fluoride application, but as such application is usually not limited to one but all posterior interdental areas, costs were calculated as 1/12 of the factorized item-fee. Effects of uncertainty emanating from this assumption were controlled in a separate scenario. Since one of the trials [10] performed resin infiltration in addition to non-invasive means, we added regular costs for topical fluoridation to this treatment arm as well. For all follow-up treatments, costs of dental diagnostics (assessment and advice, radiographs) were included within the course of treatment.

To compare cost-effectiveness of non- and micro-invasive treatment, we performed Monte-Carlo microsimulations. To allow the introduction of parameter uncertainty, we randomly sampled transition probabilities from a triangular distribution between 95% CI [20]. The effects of parameter uncertainty regarding the efficacy of micro-invasive treatment were explored using best- and worst-case scenarios based on evidence-based 95% CI. Additionally, we performed univariate sensitivity analyses to explore the effects of varying patient’s age, the social discounting rate and the assumed underlying distribution of probabilities between 95% CI. Mean point-estimates for costs (c, in Euro) and effectiveness (e, in % of non-restored teeth) as well as incremental cost-effectiveness ratios (ICER = Δc/Δe) were calculated [21]. The net benefit of each treatment was calculated using the formula NB = λ×Δe−Δc [22], with λ denoting the ceiling threshold of willingness to pay, *i.e.* the additional costs a decision maker is willing to sacrifice for gaining an additional unit of effectiveness [21]. If λ>Δc/Δe, an alternative intervention (micro-invasive treatment) is considered more cost-effective than the comparator (non-invasive treatment), despite possibly being more costly [20]. Using this approach, we plotted the probability of being cost-effective against different λ.

To compare the lifetime-costs of non-, micro- and invasively treatment of lesions, we performed a cost-analysis using the described Monte-Carlo microsimulations, with random sampling of transition probabilities. Additionally, a high-cost scenario was analysed to determine whether assigning full item-costs for fluoridation as well as higher factoring of item-points for initial treatments changed our estimates.

## Results

Age- and time-dependent hazard functions were used to introduce transition probabilities of non- and micro-invasively treated lesions into the model (Fig. S1, Table S1). Transition probabilities from other health states were calculated for three time-dependent plateaus (Tables S2, S3). Costs per course of treatment were calculated by quantification of chargeable items and factoring (Table S4). A summary of transition probabilities and cost estimates is shown in Tables 1, 2.

**Table 1:** Transition probabilities (p) used within the model.

State	Transition probability (p) per cycle	Transition/allocation to	Probability
**Non-invasive E2 (I)**	p = 3.0984×(2α)^−1.343^ (95% CI: p×0.87– p×1.13)	D1	1.00
**Non-invasive D1 (I)**	p = 1.652×(2α)^−2.078^ (95% CI: p×0.87– p×1.13)	Composite	1.00
**Infiltrated E2 (I)**	p = 0.4289×(2α)^−1.391^ (95% CI: p×0.23– p×5.15)	Infiltrated D1	1.00
**Infiltrated D1 (I)**	p = 68.869×(2α)^−2.078^ (95% CI: p×0.23– p×4.17)	Composite	1.00
**Composite (I/F)**	range p = 0.011–0.019	Composite	0.45
		Crown	0.10
		Repair	0.10
		RCT	0.25
		Extraction	0.10
**Direct capping (F)**	range p = 0.008–0.168	RCT	0.95
		Extraction	0.05
**Crown on vital tooth (F)**	range p = 0.019–0.041	RCT	0.25
		Recementation	0.15
		Repair	0.10
		Re- crown	0.40
		Extraction	0.10
**Root canal treatment (F)**	range p = 0.014–0.022	Non-surgical re-treatment	0.20
		Surgical re-treatment	0.30
		Extraction	0.50
**Crown on non-vital tooth (F)**	range p = 0.015–0.328	Recementation	0.20
		Repair	0.10
		Re- crown	0.60
		Extraction	0.10
**Non-surgical (F)**	range p = 0.013–0.117	Surgical re-treatment	0.25
		Extraction	0.75
**Surgical (F)**	range p = 0.015–0.065	Extraction	1.00
**Implant (F)**	range p = 0.001–0.015	Recementation/Refixing	0.60
		Re-crown	0.20
		Re-implant	0.20

Teeth were allocated to their initial health state (I) depending on the treatment strategy and the lesion stage (left column). For non- and micro-invasively treated lesions, transition probabilities per 6-monthly cycle depended on patient’s age (α) and were calculated using hazard functions (middle column). For all follow-up states (F), transition probabilities depended on the time spent in the health state (e.g. the time since a crown had been placed), with three time plateaus being modelled (<2, 2–5, >5 years). To introduce joint parameter uncertainty, a triangular distribution of parameters between their 95% Confidence Intervals (CI) was assumed. For hazard functions, 95% CI (given in brackets) were used within scenario analyses. To simplify the table, we only present the range of follow-up transition probabilities used within the model. Full details (time-dependent mean and 95% CI probabilities) can be found within the Supporting Information. If transition occurred, teeth were allocated to follow-up states according to allocation probabilities (right columns).

**Table 2:** Cost estimation.

Course of treatment	Costs	Costs
	Base-case scenario (€)	High-cost scenario (€)
Topical fluoridation^1^	0.54	9.84
Resin infiltration^1^	84.99	129.33
Composite restoration^1, 2^	92.66	130.19
Re-treatment with composite^2^	129.54	129.54
Repair of existing restoration	86.99	86.99
Direct capping and composite restoration^2^	134.88	134.88
Root canal treatment^3^	283.19	283.19
Full-metal crown	345.23	345.23
Re-cementation of a crown	54.29	54.29
Non-surgical root canal re- treatment^3^	790.64	790.64
Surgical root canal re-treatment	154.63	154.63
Tooth removal	67.41	67.41
Implant insertion	958.40	958.40
Implant-supported porcelain-bonded crown	848.27	848.27

1 Costs for dental diagnostics (items 01, 8, Ä925) not included.

2 Two-surface restoration assumed.

3 Treatment of three root canals per tooth assumed.

For each course of treatment, costs were calculated by quantification of item-fees from public or private item-catalogues (for details see Supporting Information). Within the base-case scenario, non-invasive treatment accrued costs of 1/12 item-fee for fluoridation, since we assumed that all posterior interdental areas would be fluoridated. Within the high-costs scenario, non-invasive treatment generated full costs for topical fluoridation, and a higher fee-multiplicator (×3.5) was used for factorable items of the initial therapy to reflect cost-variability. Future costs were discounted with 3% per annum.

**Figure 2:**
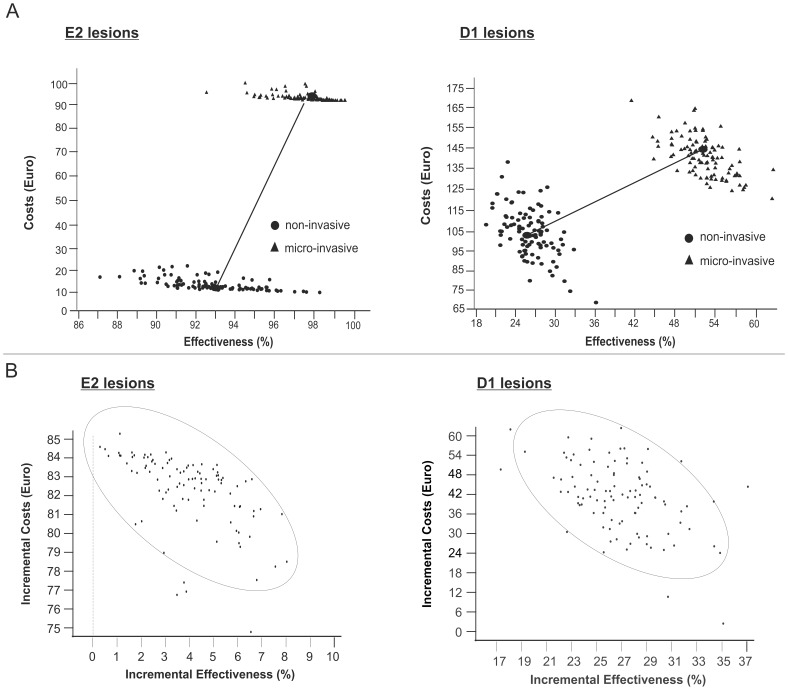
Cost-effectiveness of different treatment strategies. 2a: Cost-effectiveness-planes of non- and micro-invasive treatment of E2 (left) and D1 lesions (right). Horizontal and vertical axes represent effectiveness (% of unrestored lesions over lifetime) and lifetime treatment costs (€), respectively. For non-invasive and follow-up treatments, parameter uncertainty was introduced by random sampling from a triangular distribution within the 95% Confidence Interval. Effects of uncertainty related to micro-invasive treatment were explored using scenario analyses (see Table 3). Non-invasive treatment was less costly and less effective than micro-invasive treatment. Regardless of the initial treatment, progression of E2 lesions occurred at later stages of life and in only few lesions, with low costs for such late re-treatment due to discounting effects. Micro-invasive treatment prevented progression of an additional 4.7% of E2 lesions compared with non-invasive treatment. The low effectiveness gain at high additional costs made micro-invasive treatment less cost-effective for E2 lesions. D1 lesions had higher transition probabilities after both treatments than E2 lesions. Micro-invasive treatment prevented the progression of an additional 27.0% of D1 lesions compared with non-invasive treatment, resulting in a more pronounced effectiveness advantage. 2b: Incremental cost-effectiveness planes. Horizontal and vertical axes illustrate the effectiveness- and cost-differences between micro- compared with non-invasive treatment. The ellipses represent 95% confidence intervals. Micro-invasive treatment was more costly and effective than non-invasive therapy for both E2 (left) and D1 lesions (right). Consequently, all ICERs are found in the north-eastern quadrant. Cost-differences were higher for E2 lesions, whilst effectiveness-differences were higher for D1 lesions.

**Figure 3:**
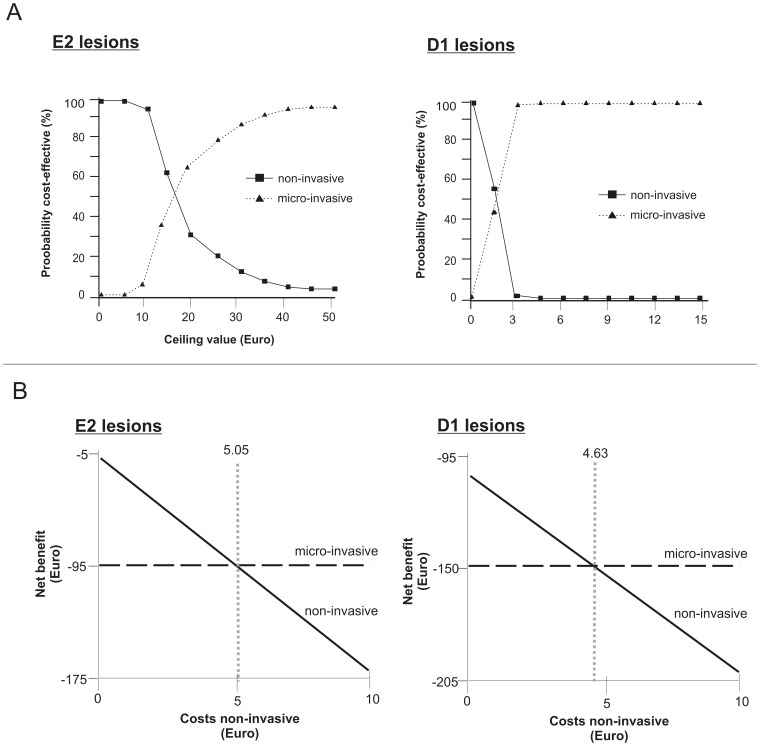
Cost-acceptability and net-benefit of different treatment strategies. 3a: Cost-effectiveness-acceptability curves. For each strategy, the probability of being cost-effective is plotted against a ceiling value (€). This value reflects the maximum a decision-maker is willing to invest to achieve an additional unit of effectiveness [20]. By increasing the ceiling value, the higher initial treatment costs of micro-invasive therapy become less important and its probability of cost-effectiveness increases. For E2 lesions, both non- and micro-invasive treatment were found to have an equal chance of cost-effectiveness at a threshold of 16.73€. Below this ceiling value, non-invasive treatment would be more likely to be cost-effective, whilst micro-invasive treatment has a higher probability of being cost-effective above that value. This value was reduced to 1.57 € for D1 lesions: These lesions had higher transition probabilities, and micro-invasive treatment prevented progression of considerably more D1 than E2 lesions (27.0% compared with 4.7%) in comparison with non-invasive treatment. This increased effectiveness resulted in a lower ceiling value threshold for D1 compared to E2 lesions. 3b: Net benefit curves. Net benefit of non- and micro-invasive treatment for E2 (left) and D1 lesions (right) depending on the costs for non-invasive therapy was calculated assuming a willingness-to-pay ceiling value of 0 €. If non-invasive therapy was more costly than 5.05 € or 4.63 €, respectively, micro-invasive treatment had the higher net benefit.

Within the base-case analysis, micro-invasive treatment was found more costly and more effective than non-invasive therapy, thus being less cost-effective for both E2 and D1 lesions (Fig. 2a, b). We found smaller cost differences and more pronounced effectiveness advantages for treating D1 lesions by micro-invasive compared to non-invasive means. Using the net-benefit approach, we calculated ceiling value thresholds of 16.73 € and 1.57 € for E2 and D1 lesions, respectively, i.e. a decision maker who is willing to sacrifice more than these monetary values for an additional effectiveness unit will find micro-invasive treatment more likely to be cost-effective than non-invasive treatment (Fig. 3a).

Within sensitivity analyses (Table 3),

**Table 3:** Cost-effectiveness of strategies in different scenarios.

Lesion stage	Scenario^1^	Strategy	c	e	Rank^2^	ICER^2^
			(€)	(%)	(d/u)	(Δ€/Δ%)
E2	Base-case^3^	Non-invasive	13.09	93.0	1	–
		Micro-invasive	95.09	97.9	2 (u)	16.73
	Best-case^4^	Non-invasive	13.09	93.0	1	–
		Micro-invasive	93.92	99.9	2 (u)	11.71
	Worst-case^5^	Non-invasive	13.09	93.0	1	–
		Micro-invasive	109.49	78.5	2 (d)	−6.65
	Age 15 years^6^	Non-invasive	13.61	91.5	1	–
		Micro-invasive	96.00	96.7	2 (u)	15.84
	Age 40 years^7^	Non-invasive	10.99	98.0	1	–
		Micro-invasive	93.90	99.7	2 (u)	48.77
	1% discount rate	Non-invasive	42.76	93.0	1	–
		Micro-invasive	114.78	97.9	2 (u)	14.70
	5% discount rate	Non-invasive	7.32	93.0	1	–
		Micro-invasive	90.79	97.9	2 (u)	17.03
	Uniform distribution	Non-invasive	13.21	93.4	1	–
		Micro-invasive	95.09	98.1	2 (u)	17.42
D1	Base-case^3^	Non-invasive	105.42	25.9	1	–
		Micro-invasive	147.74	52.9	2 (u)	1.57
	Best-case^4^	Non-invasive	105.42	25.9	1	–
		Micro-invasive	106.02	98.9	2 (u)	0.01
	Worst-case^5^	Non-invasive	105.42	25.9	1	–
		Micro-invasive	227.91	11.1	2 (d)	−8.28
	Age 15 years^6^	Non-invasive	136.10	14.2	1	–
		Micro-invasive	170.59	38.6	2 (u)	1.41
	Age 40 years^7^	Non-invasive	44.67	68.6	1	–
		Micro-invasive	110.93	84.3	2 (u)	0.80
	1% discount rate	Non-invasive	275.55	25.9	2 (d)	−0.50
		Micro-invasive	262.13	52.9	1	–
	5% discount rate	Non-invasive	65.61	25.9	1	–
		Micro-invasive	122.16	52.9	2 (u)	2.09
	Uniform distribution	Non-invasive	106.32	27.1	1	–
		Micro-invasive	147.74	53.3	2 (u)	1.58

1 Input data regarding effectiveness within scenarios taken from Table 1.

2 Calculated to highest ranked strategy. Negative values indicate additional costs per effectiveness loss; positive values indicate additional costs per effectiveness gain. Strategies were either dominated (d) or undominated (u) by the first-ranked strategy.

3 Base-case: 20-year-old-patient with 58.25 years to live [19]; replacement of 80% of removed teeth assumed [26]; 3% discounting rate [18], triangular distribution of probabilities between 95% CI assumed.

4 Best-case: Highest evidence-based effectiveness of micro-invasive treatment assumed.

5 Worst-case: Lowest evidence-based effectiveness of micro-invasive treatment assumed.

6 Years to live: 63.5 [19].

7 Years to live: 39.0 [19].

Mean costs (c, €) and effectiveness (e, % of unrestored lesions), ranking of strategies as well as incremental cost-effectiveness ratios (ICERs) were calculated. Ranking was performed according to costs (strategies with higher costs were ranked lower). Cost-effectiveness analyses were performed separately for lesions of different stages (E2 or D1). Besides the base-case analysis, we performed best- and worst-case sensitivity analyses to explore effects of uncertainty resulting from current evidence. Within these analyses, we varied the transition probabilities of micro-invasively treated lesions based on the 95% CI of calculated Risk Ratios of our meta-analysis. We additionally explored the effects of the patient’s age as well as used discount rates and applied distribution of probabilities for random sampling on the cost-effectiveness estimates.

micro-invasive treatment was dominated in both worst-case scenarios,
the cost-effectiveness of micro- compared to non-invasive treatment was decreasing with increasing patient’s age,
increasing the discount rate to 5% did not considerably change our estimates,
decreasing the discount rate to 1% led to domination of micro- over non-invasive treatment of D1 lesions,
assuming a uniform instead of a triangular distribution of probabilities did not considerably affect cost-effectiveness,
given a willingness-to-pay of 0 €, non-invasive treatment was assumed to have a lower net benefit than micro-invasive treatment, if its costs exceeded 5.05 € and 4.63 € for E2 and D1 lesions, respectively, (Fig. 3b).

**Figure 4:**
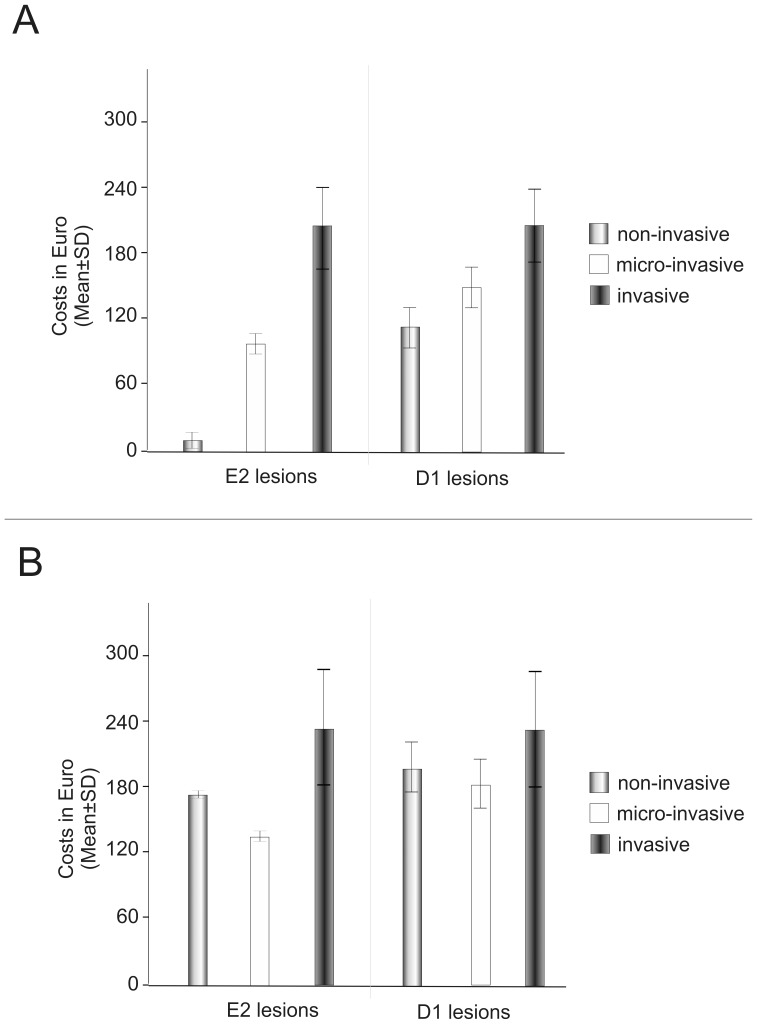
Lifetime costs of different treatment strategies. 4a: Costs were analysed within the base-case scenario (20-year old patient, life expectancy 58.25 years, discount rate 3% per year, initial treatment costs for non-, micro- and invasive treatment 0.54 €, 84.99 € and 92.66 €, respectively). Costs for invasively treated lesions were not influenced by lesion stage. Since E2 lesions had lower transition probabilities than D1 lesions, lifetime costs for non- or micro-invasively treated E2 lesions were reduced compared to D1 lesions. Due to reduced efficacy of non-invasive treatment for D1 lesions, the cost-advantage of non-invasive compared to micro-invasive treatment was considerably reduced for these lesions. Invasive treatment was the most expensive option for both E2 and D1 lesions. 4b: Lifetime costs within the high-cost scenario. Non-invasive was assumed to accrue costs of 9.84 € for topical fluoridation each cycle, followed by costs for follow-up treatments. Micro-invasive treatment initially generated costs of 129.33 €, followed by regular costs for topical fluoridation and all follow-up treatments. Invasive treatment was assumed to initially generate costs of 130.19 €, followed by costs for follow-up treatment. Within this scenario, micro-invasive treatment was the least costly treatment for both E2 and D1 lesions.

The comparison of the lifetime costs of non-, micro- and invasive treatment of E2 lesions revealed significant differences between the three methods, with non-invasive therapy being the least costly strategy, followed by micro-invasive treatment. These differences were less pronounced for D1 lesions (Fig. 4a). Within the high-cost scenario, micro-invasive treatment was the least costly strategy for both E2 and D1 lesions, followed by non- and invasive treatment (Fig. 4b).

## Discussion

Non- and micro-invasive therapies aim at delaying restorative measures to reduce or avoid the cycle of re-treatments, which compromises the integrity and survival of the tooth [6], [7]. The delay of this cycle might have the biggest effect in patients with high risk, which are – due to the skewed distribution of caries lesions within most societies – also the neediest and possibly most cost-sensitive patients [23]. Within the present study, both non- and micro-invasive treatment were found suitable to delay re-treatments, with micro-invasive treatment being more effective but also generally more costly than non-invasive therapy. The effectiveness advantages of micro-invasive treatment were increased for the treatment of D1 compared with E2 lesions. This was unexpected, since the efficacy of micro-invasive treatment was shown to be lower for D1 than E2 lesions [14], [15]. However, D1 lesions are progressing significantly faster than E2 lesions [5], [12], [13]. Thus, infiltration leads to a high relative reduction of progression speed of E2 lesions, but the absolute reduction is higher in D1 than E2 lesions.

Besides lesion stage, the patient’s age influenced the cost-effectiveness estimates. Increasing the patient’s age reduced the effectiveness advantage of micro- compared to non-invasive treatment. This was due to the lower progression risk of non-invasively lesions in older compared to younger patients, and the shorter remaining lifetime of older patients (with less chance to accumulate high re-treatment costs). We did, however, not account for the age of the patient when calculating the risk ratios of micro- versus non-invasively treated E2 or D1 lesions. Since older patients are likely to have less active lesions [12], with reduced chance of sufficient infiltration, it is likely that age has even greater influence on effectiveness under clinical conditions. In conclusion, micro-invasive treatment seems most beneficial for young patients with D1 lesions.

If only costs were analysed, non-invasive treatment was significantly less costly than other options for treating E2 lesions, since only few of these lesions progress, which reduces the need for costly re-interventions. Thus, the initial treatment costs are the main component in total lifetime costs of non- and micro-invasively treated E2 lesions. In contrast, D1 lesions progress more swiftly and require expensive re-treatment earlier. This reduces the importance of the initial treatment costs and levelled the differences between the initially cheap non-invasive and initially expensive micro-invasive treatment. In the base-case scenario, non-invasive therapy itself was assumed to generate only small costs, since measures like topical fluoridation are usually not limited to only one tooth. In addition, we assumed that infiltrated lesions may be additionally fluoridated as well, since this was done in one of the analysed clinical trials. However, in some healthcare settings fluoridation etc. will generate tooth-based costs every time it is performed. This was simulated in the high-costs scenario, with regular generation of full costs for fluoridation. In this scenario, our cost-rankings were changed, with micro-invasive treatment being the least costly strategy for both E2 and D1 lesions. We calculated cost threshold values of around 5 € for non-invasive therapy, i.e. above these costs micro-invasive treatment has a higher chance of being cost-effective than non-invasive treatment. Cost estimates were further shown to depend on the discount rate, with higher discounting decreasing the cost-effects of late, expensive re-treatment.

Based on the best available evidence regarding underlying effectiveness parameters, our findings were found to be robust. However, evidence levels are still limited and further research may change our estimates. Several studies regarding caries infiltration have been performed but not published or used within our meta-analysis: A practice-based study in Germany found a radiographic progression of 3/96 infiltrated lesions (3%) and 23/96 non-invasively treated lesions (22%) (HML, personal communication). Similarly, the 5-year-follow-up results from one included study found 2/19 infiltrated (11%) and 10/19 (53%) non-invasively treated lesions to progress (SP, personal communication). Another study confirmed these findings for deciduous teeth [24]. One disadvantage of the included studies is their setting in university hospitals. Trials there may overestimate the advantages of micro- compared with non-invasive treatment in general practice, since patient selection and treatment conditions in a university hospital may be different than in a primary care environment. However, the practice-based study reported nearly congruent preventive fractions when compared to university studies, and given the low heterogeneity of the results of our meta-analysis, the effects of the setting on our estimates should be limited.

There are certain limitations of our study. We did not account for differences of gender or life-expectancy. Given the moderate influence of modeled heterogeneity, we do not expect such parameters to change our estimates substantially. Similarly, we introduced uncertainty based on random sampling of probabilities from a triangular distribution between 95% CI. This exact distribution is unlikely in reality. However, we did not find our estimates greatly altered if a uniform distribution was assumed, and effects of distribution on calculated rankings are probably limited. Similarly, we did not account for possible within-patient correlations (patients with higher caries risk will have faster lesion progression and may also have increased risk of failure of restorations etc.). It would be of great interest to analyze the cost-effectiveness of micro- compared with non-invasive treatment in different risk groups. However, a published study investigating infiltration in children with high caries risk did not indicate lower efficacy of infiltration in such patients [24]. One further issue is the assumption of a standard of non-invasive care. Non-invasive measures have changed over the last decades and patients’ adherence cannot always be assumed. Thus, the effectiveness of non-invasive means may be altered, which could affect our estimates. Lastly, our estimates were calculated for posterior proximal surfaces irrespective of tooth type or surface. Progression risks of proximal caries as well as risk of failures of restorations differ between molars and premolars or mesial and distal surfaces [5], [12], [25]. Thus, cost and effectiveness parameters may vary on the tooth- and the surface-level. Similarly, treatment of primary teeth will result in different cost-effectiveness estimates, but given the limited lifespan of primary teeth and the different applicability of treatments in the primary dentition, we decided not to model treatment of primary teeth.

In comparison with invasive therapy, both non- and micro-invasive treatment reduce the lifetime costs associated with the treatment of proximal posterior E2 or D1 lesions. Micro-invasive treatment is more effective, but usually more costly than non-invasive therapy. Micro-invasive therapy had its highest cost-effectiveness for treating D1 lesions in young patients. Decision makers with a willingness-to-pay a minimum of 16.73 € for E2 and 1.57 € for D1 lesions, respectively, will find micro-invasive treatment more cost-effective than non-invasive therapy. Based on current evidence and within the characteristics of the German healthcare setting, our findings were robust regarding uncertainty and heterogeneity.

## Supporting Information

Figure S1 **Meta-analysis of micro- versus non-invasive treatment.** Study data, weighted Risk Ratios (RR) and 95% Confidence Intervals (95%CI), heterogeneity I^2^ and overall effect statistics as well as Forest plots are shown. Analyses were performed separately for progression risk from E2 to D2 and D1 to any deeper lesion.
(DOC)

Table S1 **Risk of bias.**
(DOCX)

Table S2 **Transition probabilities and hazard functions for each health state.** Depending on the patient’s age and the time since the last treatment, transition probabilities were calculated or hazard functions used for modelling of probabilities. Mean and 95% Confidence Intervals (95% CI) are given, 95% CI were used for modelling scenarios or to allow random sampling of probabilities. The source on which calculation of each probability is based is given. Allocation was decided based on current evidence and the decision of an expert panel.
(DOC)

Table S3 **Meta-analysis 2.** Since we did not find a suitable meta-analysis for annual failure after direct capping, this meta-analysis was performed. Mean and 95% CI were calculated and introduced within the model (see table S2).
(DOC)

Table S4 **Costs.**
(DOCX)

Text S1 **References for Supporting Information.**
(DOCX)
