# Supplementary material for: Costs and Effectiveness of Treatment Alternatives for Proximal Caries Lesions
Source: PLoS One. 2014 Jan 27;9(1):e86992. doi: 10.1371/journal.pone.0086992 (PMC3903601; doi:10.1371/journal.pone.0086992)
Supplement: Figure S1 — Meta-analysis of micro- versus non-invasive treatment. Study data, weighted Risk Ratios (RR) and 95% Confidence Intervals (95%CI), heterogeneity I2 and overall effect statistics as well as Forest plots are shown. Analyses were performed separately for progression risk from E2 to D2 and D1 to any deeper lesion. (DOC) [file pone.0086992.s001.doc]

Supplementary Figure S1: Meta-analysis of micro- versus non-invasive treatment. Study data, weighted Risk Ratios (RR) and 95% Confidence Intervals (95%CI), heterogeneity I^2^ and overall effect statistics as well as Forest plots are shown. Analyses were performed separately for progression risk from E2 to D2 and D1 to any deeper lesion.

| **Outcome** | **Study** | **Events** | **Total** | **Events** | **Total** | **Weight** | **Risk Ratio [95% CI]** | | **Risk Ratio (95%CI)** | | |  | | | | | |
| --- | --- | --- | --- | --- | --- | --- | --- | --- | --- | --- | --- | --- | --- | --- | --- | --- | --- |
| **Progression  E2 🡪 D1** |  |  |  |  |  |  |  |  | |  | | | | | | | |
|  | [Martign*on et a*l., 2012](#_ENREF_1) | 1 | 15 | 7 | 14 | 67.0% | 0.13 [0.02, 0.95] |  | |  | | | | | | | |
|  | [Par*is et a*l., 2010](#_ENREF_2) | 0 | 11 | 5 | 17 | 33.0% | 0.14 [0.01, 2.25] |  | |  | | | | | | | |
|  |  |  |  |  |  |  |  |  |  |  |  |  |  |  |  |  |  |
|  | **Total (95% CI)** |  | **26** |  | **31** | **100.0%** | **0.13 [0.03, 0.67]** |  | | |  | | | | | | |
|  | **Total events** | **1** |  | **12** |  |  |  |  | | |  | | | | | | |
|  |  |  |  |  |  |  |  |  |  |  |  |  |  |  |  |  |  |
|  | Heterogeneity: Tau² = 0.00; Chi² = 0.00, df = 1 (P = 0.99); I² = 0% | | | | | |  | 0.01 0.1 0.0 10 100 favours infiltration favours control | |  | | | | | | | |
|  | Test for overall effect: Z = 2.45 (P = 0.01) | | | | | | |  | | |  | |  |  |  |  | Favours infiltration Favours control |
| **Progression  D1 🡪 any** |  |  |  |  |  |  |  |  | |  | | | | | | | |
|  | [Martign*on et a*l., 2012](#_ENREF_1) | 11 | 22 | 17 | 23 | 69.9% | 0.68 [0.42, 1.10] |  | |  | | | | | | | |
|  | [Par*is et a*l., 2010](#_ENREF_2) | 1 | 15 | 4 | 9 | 30.1% | 0.15 [0.02, 1.14] |  | |  | | | | | | | |
|  |  |  |  |  |  |  |  |  |  |  |  |  |  |  |  |  |  |
|  | **Total (95% CI)** |  | **37** |  | **32** | **100.0%** | **0.43 [0.10, 1.80]** |  | | |  | | | | | | |
|  | **Total events** | **12** |  | **21** |  |  |  |  | | |  | | | | | | |
|  |  |  |  |  |  |  |  |  |  |  |  |  |  |  |  |  |  |
|  | Heterogeneity: Tau² = 0.70; Chi² = 2.24, df = 1 (P = 0.13); I² = 55% | | | | | |  | 0.01 0.1 0.0 10 100 favours infiltration favours control | |  | | | | | | | |
|  | Test for overall effect: Z = 1.15 (P = 0.25) | | | | | | |  | | |  | | | | | | |
