# Supplementary material for: Costs and Effectiveness of Treatment Alternatives for Proximal Caries Lesions
Source: PLoS One. 2014 Jan 27;9(1):e86992. doi: 10.1371/journal.pone.0086992 (PMC3903601; doi:10.1371/journal.pone.0086992)
Supplement: Table S1 — Risk of bias. (DOCX) [file pone.0086992.s002.docx]

Supplementary table S1: Risk of bias, assessed according to Cochrane guidelines ([Higgins and Green, 2011](#_ENREF_9)).

|  | Random sequence generation (selection bias) | Allocation concealment (selection bias) | Blinding of participants and personnel (performance bias) | Blinding of outcome assessment (detection bias) | Incomplete outcome data addressed (attrition bias) | Selective reporting (reporting bias) |
| --- | --- | --- | --- | --- | --- | --- |
| Paris and Meyer-Lueckel 2010 | + | + | + | + | + | + |
| Martignon et al., 2013 | + | ? | - | + | + | + |
