# Supplementary material for: Costs and Effectiveness of Treatment Alternatives for Proximal Caries Lesions
Source: PLoS One. 2014 Jan 27;9(1):e86992. doi: 10.1371/journal.pone.0086992 (PMC3903601; doi:10.1371/journal.pone.0086992)
Supplement: Table S2 — Transition probabilities and hazard functions for each health state. Depending on the patient’s age and the time since the last treatment, transition probabilities were calculated or hazard functions used for modelling of probabilities. Mean and 95% Confidence Intervals (95% CI) are given, 95% CI were used for modelling scenarios or to allow random sampling of probabilities. The source on which calculation of each probability is based is given. Allocation was decided based on current evidence and the decision of an expert panel. (DOC) [file pone.0086992.s003.doc]

Supplementary table S2: Transition probabilities and hazard functions for each health state. Depending on the patient’s age and the time since the last treatment, transition probabilities were calculated or hazard functions used for modelling of probabilities. Mean and 95% Confidence Intervals (95% CI) are given, 95% CI were used for modelling scenarios or to allow random sampling of probabilities. The source on which calculation of each probability is based is given. Allocation was decided based on current evidence and the decision of an expert panel.

| State (Age of patient) | Source | Probability (p) and distribution (95% CI%) of re-treatment per cycle after | | | | | | Transition to | Prob. | Remarks |
| --- | --- | --- | --- | --- | --- | --- | --- | --- | --- | --- |
|  |  | 0-2 years | | 2-5 years | | >5 years | |  |  |  |
|  |  | p | 95% CI | p | 95% CI | p | 95% CI |  |  |  |
| Non-invasive E2 (<16) | [Mejà*re et a*l., 2004](#_ENREF_13) | Depending on patient’s age (a): p = 3.0984×(2a)^-1.343^  Distribution: p*0.87 – p*1.13 | | | | | | D1 lesion | 1.00 |  |
| Non-invasive E2 (16-19) |  |  |  |  |  |  |  |  |  |  |
| Non-invasive E2 (>19) |  |  |  |  |  |  |  |  |  |  |
| Non-invasive D1(<16) |  | Depending on patient’s age (a): p = 1.652×(2a)^-2.078^  Distribution: p*0.87 – p*1.13 | | | | | | Composite | 1.00 |  |
| Non-invasive D1 (16-19) |  |  |  |  |  |  |  |  |  |  |
| Non-invasive D1 (>19) |  |  |  |  |  |  |  |  |  |  |
| Infiltrated E2 (<16) | Meta-analysis 1 | Depending on patient’s age (a): p = 0.4289×(2a)^-1.391^  Distribution: p*0.23 – p*5.15 | | | | | | Infiltrated D1 | 1.00 | Based on pair-wise analysis. Only progression into the next stage was used for calculation. RR (95% CI) 0.13 (0.03-0.67) (table S2). |
| Infiltrated E2 (16-19) |  |  |  |  |  |  |  |  |  |  |
| Infiltrated E2 (>19) |  |  |  |  |  |  |  |  |  |  |
| Infiltrated D1(<16) |  | Depending on patient’s age (a): p = 68.869×(2a)^-2.078^  Distribution: p*0.23 – p*4.17 | | | | | | Composite | 1.00 | Based on pair-wise analysis. Any progression was used for calculation. Based on RR (95% CI) 0.43 (0.1-1.8) (table S1). |
| Infiltrated D1 (16-19) |  |  |  |  |  |  |  |  |  |  |
| Infiltrated D1 (>19) |  |  |  |  |  |  |  |  |  |  |
| Composite | [Palles*en et a*l., 2013](#_ENREF_16) | 0.016 | 0.014-0.019 | 0.016 | 0.014-0.019 | 0.012 | 0.011-0.015 | Composite  Crown  Repair  RCT  Extraction | 0.45  0.10  0.10  0.25  0.10 | Data from 15-19-year-olds. For 0-5 years, linear failure rate assumed. >5 assumed on 5-year basis. Allocation based on [Opd*am et a*l., 2010](#_ENREF_15). Risk of pulpal exposure during re-composite assumed to be 10%. Crowning only assumed if already re-restored before, instead repair assumed more often. |
| Direct capping | Tab. S3 | 0.111 | 0.054-0.168 | 0.064 | 0.022-0.145 | 0.031 | 0.008-0.075 | RCT  Extraction | 0.95  0.50 |  |
| Crown on vital tooth | [Burke and Lucarotti, 2009](#_ENREF_3) | 0.036 | 0.031-0.041 | 0.027 | 0.022-0.038 | 0.024 | 0.019-0.036 | RCT  Recementation  Repair  Re- crown  Extraction | 0.25  0.15  0.10  0.40  0.10 | Used range of crowns for uncertainty. For non-vital crowned teeth, risk of endodontic complications was calculated separately and AFR of crown itself reduced according to [Ferra*ri et a*l., 2012](#_ENREF_6). |
| Root canal and | [Luml*ey et a*l., 2008](#_ENREF_10) | 0.021 | 0.022-0.020 | 0.017 | 0.018-0.016 | 0.015 | 0.014-0.016 | Non-surgical re-treatment Surgical re-treatment Extraction | 0.20  0.30  0.50 |  |
| Crown on non-vital tooth | [Burke and Lucarotti, 2009](#_ENREF_3) | 0.0288 | 0.0248-0.0328 | 0.0216 | 0.0176-0.0304 | 0.0192 | 0.0152-0.0288 | Recementation  Repair  Re- crown  Extraction | 0.20  0.10  0.60  0.10 | Used range of crowns for uncertainty. Risk of endodontic complications was calculated separately and AFR reduced according to [Ferra*ri et a*l., 2012](#_ENREF_6). |
| Non-surgical | [*Ng et a*l., 2008](#_ENREF_14) | 0.085 | 0.057-0.117 | 0.019 | 0.013-0.026 | 0.019 | 0.013-0.026 | Surgical re-treatment  Extraction | 0.25  0.75 | >5 assumed on 5-year basis |
| Surgical | [Torabinej*ad et a*l., 2009](#_ENREF_20) | 0.061 | 0.057-0.065 | 0.033 | 0.030-0.035 | 0.023 | 0.015-0.028 | Extraction | 1.00 | Weighted used. >5 assumed on 10-year basis |
| Implant | [Torabinej*ad et a*l., 2007](#_ENREF_19) | 0.010 | 0.008-0.015 | 0.003 | 0.002-0.005 | 0.002 | 0.001-0.002 | Recementation/Refixing Re-crown  Re-implant | 0.60  0.20  0.20 | >5 assumed on 10-year basis |
