# Supplementary material for: Costs and Effectiveness of Treatment Alternatives for Proximal Caries Lesions
Source: PLoS One. 2014 Jan 27;9(1):e86992. doi: 10.1371/journal.pone.0086992 (PMC3903601; doi:10.1371/journal.pone.0086992)
Supplement: Table S3 — Meta-analysis 2. Since we did not find a suitable meta-analysis for annual failure after direct capping, this meta-analysis was performed. Mean and 95% CI were calculated and introduced within the model (see table S2). (DOC) [file pone.0086992.s004.doc]

Supplementary table S3: Meta-analysis 2. Since we did not find a suitable meta-analysis for annual failure after direct capping, this meta-analysis was performed. Mean and 95% CI were calculated and introduced within the model (see table S2).

| **Direct capping** | ≤2 years | 2-5 years | >5 years |
| --- | --- | --- | --- |
| [C*ho et a*l., 2013](#_ENREF_4) | 26.1% | 15.8% |  |
| [Fitzgerald and Heys, 1991](#_ENREF_7) | 25.0% |  |  |
| [Mats*uo et a*l., 1996](#_ENREF_12) | 16.0% |  |  |
| [Al-Hiyas*at et a*l., 2006](#_ENREF_1) |  | 13.4% |  |
| [Barth*el et a*l., 2000](#_ENREF_2) |  | 8.8% | 7.9% |
| [Shovelt*on et a*l., 1971](#_ENREF_18) | 15.0% |  |  |
| [Dammasch*ke et a*l., 2010](#_ENREF_5) | 10.0% | 4.0% | 1.5% |
| [Willershaus*en et a*l., 2011](#_ENREF_21) | 20.0% | 6.4% | 4.6% |
| **Mean (95% CI)** | **18.7 (10.0/26.0)%** | **9.7  (4.0/15.8)%** | **4.7**  **(1.5/7.9)%** |
