# Supplementary material for: Costs and Effectiveness of Treatment Alternatives for Proximal Caries Lesions
Source: PLoS One. 2014 Jan 27;9(1):e86992. doi: 10.1371/journal.pone.0086992 (PMC3903601; doi:10.1371/journal.pone.0086992)
Supplement: Table S4 — Costs. (DOCX) [file pone.0086992.s005.docx]

Supplementary table S4: Costs

Patients were assumed to be enrolees of statutory German health insurance (“social health insurance”), which covers 88% of the total population ([GKV-Spitzenverband, 2013](#_ENREF_8)). We did not calculate costs separately for privately insured patients. Additional private fees for some treatments (caries infiltration, posterior composites, re-root-canal-treatments, implant-retained crowns) are paid by the patient.

**Calculation of Euro *per* point**

The German healthcare system is based on item-fee-points, with different fee catalogues for different treatment procedures and patients.

| **Federal state** | **Euro/point** |
| --- | --- |
| Baden-Wurttemberg | 0.9354 |
| Bayern | 0.8861 |
| Berlin | 0.8447 |
| Brandenburg | 0.8614 |
| Bremen | 0.8601 |
| Hamburg | 0.9500 |
| Hessen | 0.9030 |
| Mecklenburg-Vorpommern | 0.8332 |
| Niedersachsen | 0.8886 |
| Nordrhein | 0.9145 |
| Rheinland-Pfalz | 0.8689 |
| Saarland | 0.8804 |
| Sachsen | 0.8154 |
| Sachsen | 0.8469 |
| Schleswig-Holstein | 0.9500 |
| Thüringen | 0.9019 |
| Westfalen-Lippe | 0.9410 |
| **Mean (rounded)** | **0.8900** |

*BEMA* (public insurance)

BEMA points vary between federal states, insurers and treatment groups. For our cost calculation we used mean state points for the biggest insurer (AOK) to calculate non-weighted means.

Conservative or surgical dentistry: 0.890 Euro/point
(see table on the right)

Prosthetic dentistry: 0.7771 Euro/point (fixed for all states)

*GOZ* (private insurance or private payer)

0.0562421 Euro/point (fixed)

*GOÄ* (public insurance)

9 GOÄ points equal 1 BEMA point.

We calculated BEMA points in the following tables.

*BELII/BEB* (laboratory costs public/private insurance)

Laboratory and material costs were estimated based on Laboratory Fee Catalogues (BEL II/BEB). Costs for BELII/BEB have been transformed into monetary values for the following tables.

**Calculation of costs *per* course of treatment**

Number of treatments was assumed to be ‘1’ if not indicated otherwise. For completely private treatments monetary values are given instead of points (Euro, factor x 2.3). For the initial treatments, costs for diagnostics were not included, since we assumed that such costs have occurred before the treatment (during basic screening etc.) and are thus not part of the treatment itself.

1. Fluoridation, each visit

| Treatment | Position BEMA/GOÄ/GOZ/L | Base-case Euro | High-cost Euro |
| --- | --- | --- | --- |
| Topical fluoridation per visit” | 1020 | 6.47 / 12 = 0.54 | 9.84 |
| **Total** |  | **0.54** | **9.84** |

1. Caries infiltration, per surface

| Treatment | Position BEMA/GOÄ/GOZ/L | Base-case Euro | High-cost Euro |
| --- | --- | --- | --- |
| Separation of teeth | 2030 | 8.41 | 12.80 |
| Use of rubberdam | 2040 | 8.41 | 12.80 |
| Analogue-item “adhesive restoration single surface” | 2060 | 68.17 | 103.73 |
| **Total** |  | **84.99** | **129.33** |

1. Initial composite restoration

| Treatment | Position BEMA/GOÄ/GOZ/L | Base-Case Euro | High-cost Euro |
| --- | --- | --- | --- |
| Anaesthesia | 40/41a | 11.84 | 11.84 |
| Special measurements during restorative therapy | 12 | 8.90 | 8.90 |
| Adhesive restoration, two surfaces | GOZ 2080 | 71.92 | 109.45 |
| **Total** |  | **92.66** | **130.19** |

1. Composite re-restoration

| Treatment | Position BEMA/GOÄ/GOZ/L | Points | Euro |
| --- | --- | --- | --- |
| Clinical investigation | 01 | 18 | 16.02 |
| Sensitivity testing | 8 | 6 | 5.34 |
| Radiographic assessment | GOÄ925a | 12 | 10.68 |
| Anaesthesia | 40/41a | 8/12 | 11.34 |
| Special measurements during restorative therapy | 12 | 10 | 8.90 |
| Adhesive restoration, two surfaces | GOZ 2080 | 556 | 71.92 |
| Liner | 25 | 6 | 5.34 |
| Direct capping (only for exposed pulps) | 26 | 6 | (5.34) |
| **Total** |  |  | **129.54 (134.88)** |

1. Repair of existing restorations

| Treatment | Position BEMA/GOÄ/GOZ/L | Points | Euro |
| --- | --- | --- | --- |
| Clinical investigation | 01 | 18 | 16.02 |
| Sensitivity testing | 8 | 6 | 5.34 |
| Radiographic assessment | GOÄ925a | 12 | 10.68 |
| Anaesthesia | 40/41a | 8/12 | 11.34 |
| Filling, three surfaces | 13c | 49 | 43.61 |
| **Total** |  |  | **86.99** |

1. Excavation, no direct capping, two-step excavation and temporary restoration (1st step)

| Treatment | Position BEMA/GOÄ/GOZ/L | Points | Euro |
| --- | --- | --- | --- |
| Clinical investigation | 01 | 18 | 16.02 |
| Sensitivity testing | 8 | 6 | 5.34 |
| Radiographic assessment | GOÄ925a | 12 | 10.68 |
| Anaesthesia | 40/41a | 8/12 | 11.34 |
| Special measurements during restorative therapy | 12 | 10 | 8.90 |
| Liner | 25 | 6 | 5.34 |
| **Total** |  |  | **57.62** |

1. Root canal treatment

| Treatment | Position BEMA/GOÄ/GOZ/L | Points | Number of treatments | Euro |
| --- | --- | --- | --- | --- |
| Clinical investigation | 01 | 18 |  | 16.02 |
| Sensitivity testing | 8 | 6 |  | 5.34 |
| Radiographic assessment | GOÄ925a | 12 | 3 | 32.04 |
| Anaesthesia | 40/41a | 8/12 |  | 11.34 |
| Rubberdam | 12 | 10 | 3 | 8.90 |
| Direct core build-up | 13b | 39 |  | 34.71 |
| Vital pulp exstirpation | 28 | 18 per canal | 3 | 48.06 |
| Root canal treatment | 32 | 29 per canal | 3 | 77.43 |
| Root canal filling | 35 | 17 per canal | 3 | 45.39 |
| **Total** |  |  |  | **283.19** |

1. Full metal crown

| Treatment | Position BEMA/GOÄ/GOZ/L | Points or Euro | Euro |
| --- | --- | --- | --- |
| Clinical investigation | 01 | 18 | 16.02 |
| Sensitivity testing | 8 | 6 | 5.34 |
| Radiographic assessment | GOÄ925a | 12 | 10.68 |
| Anaesthesia | 40/41a | 8/12 | 11.34 |
| Special measurements during restorative therapy | 12 | 10 | 8.90 |
| Temporary crown | 19 | 19 | 14.77 |
| Crown full metal | 20a | 148 | 115.01 |
| Dental materials |  |  | 22.07 |
| Situation model | 0010 | 5.74 | 11.48 |
| Used resin | 0023 | 12.14 | 12.14 |
| Single-tooth dye | 0051 | 9.19 | 9.19 |
| Occludator | 0120 | 8.42 | 8.42 |
| Full-metal crown | 1021 | 72.27 | 72.27 |
| Non-precious metal alloy | 9700 | 11.68 | 11.68 |
| Delivery | 9330 | 3.98 | 15.92 |
| **Total** |  |  | **345.23** |

1. Recementation

| Treatment | Position BEMA/GOÄ/GOZ/L | Points | Euro |
| --- | --- | --- | --- |
| Clinical investigation | 01 | 18 | 16.02 |
| Sensitivity testing | 8 | 6 | 5.34 |
| Radiographic assessment | GOÄ925a | 12 | 10.68 |
| Recementation of a crown | 24a | 25 | 22.25 |
| **Total** |  |  | **54.29** |

1. Non-surgical root canal retreatment

| Treatment | Position BEMA/GOÄ/GOZ/L | Points | Number of treatments | Euro |
| --- | --- | --- | --- | --- |
| Clinical investigation | 01 | 18 |  | 16.02 |
| Sensitivity testing | 8 | 6 |  | 5.34 |
| Radiographic assessment | GOÄ 5000 | 50 | 3 | 19.40 |
| Anaesthesia | GOÄ 0090/0100 | 60/70 |  | 8.41 |
| Rubberdam | GOZ 2440 | 65 | 3 | 8.41 |
| Root canal treatment | GOZ 2410 | 392 per canal | 3 | 152.09 |
| Irrigation | GOZ 2420 | 70 | 3 | 9.05 |
| Microscope | GOZ 0110 |  | 3 | 155.20 |
| Root canal filling | GOZ 2440 | 258 per canal | 3 | 100.10 |
| Medication | GOZ 2430 | 204 per visit | 3 | 52.76 |
| **Total** |  |  |  | **526.78** |

1. Surgical root canal retreatment

| Treatment | Position BEMA/GOÄ/GOZ/L | Points | Number of treatments | Euro |
| --- | --- | --- | --- | --- |
| Clinical investigation | 01 | 18 |  | 16.02 |
| Sensitivity testing | 8 | 6 |  | 5.34 |
| Anaesthesia | 40/41a | 8/12 |  | 11.34 |
| Apisectomy | 54b | 96 |  | 85.44 |
| Retrograde filling | 35 | 17 per canal |  | 15.13 |
| Radiographic assessment | GOÄ925a | 12 | 2 | 21.36 |
| **Total** |  |  |  | **154.63** |

1. Tooth removal

| Treatment | Position BEMA/GOÄ/GOZ/L | Points | Number of treatments | Euro |
| --- | --- | --- | --- | --- |
| Clinical investigation | 01 | 18 |  | 16.02 |
| Sensitivity testing | 8 | 6 |  | 5.34 |
| Radiographic assessment | GOÄ925a | 12 | 2 | 21.36 |
| Anaesthesia | 40/41a | 8/12 |  | 11.34 |
| Extraction multi root tooth | 44 | 15 |  | 13.35 |
| **Total** |  |  |  | **67.41** |

1. Implant insertion

| Treatment | | Position BEMA/GOÄ/GOZ/L | Number of treatments | Euro | | |  |  |  |
| --- | --- | --- | --- | --- | --- | --- | --- | --- | --- |
| Initial charting and consultation | | GOÄ1 |  | | 10.73 | | |  |  |
| Intraoral investigation | | GOÄ6 |  | | 13.41 | | |  |  |
| Detailed consultation | | GOÄ3 |  | | 16.31 | | |  |  |
| Cost estimation | | GOZ 0030 |  | | 25.87 | | |  |  |
| Panoramic radiograph | | GOÄ 5004 | 2 | | 116.58 | | |  |  |
| Diagnostic models | | GOZ 0050 |  | | 15.52 | | |  |  |
| Radiographic diagnosis and guide | | GOZ 9000 |  | | 114.35 | | |  |  |
| Use of radiographic guide | | GOZ 9003 |  | | 12.94 | | |  |  |
| Implant insertion | | GOZ 9010 |  | | 199.86 | | |  |  |
| Implant (Camlog, 2013) | |  |  | | 131.86 | | |  |  |
| Suture material | |  |  | | 7.68 | | |  |  |
| Post-operative care | | GOZ 3300 | 2 | | 16.82 | | |  |  |
| Prescription and medication | | GOÄ 70 |  | | 5.36 | | |  |  |
| Topical anaesthesia | | GOZ 0080 | 2 | | 3.88 | | |  |  |
| Anaesthesia | | GOÄ 0090/0100 | 2 | | 25.60 | | | |  |
| Implant re-exposure | | GOZ 9040 |  | | | 80.98 | | | |
| Gingival former | |  |  | | | 25.70 | | | |
| *Laboratory* | | | | | | | | | |
| Situation model | 0002 | | 3 | | | 28.08 | | | |
| Replica | 0241 | |  | | | 15.20 | | | |
| Occludator | 0402 | |  | | | 10.16 | | | |
| Diagnostic wax-up | 0832 | |  | | | 10.30 | | | |
| Positioning splint | 1224 | |  | | | 60.20 | | | |
| Radiographic guide | 1311 | |  | | | 3.92 | | | |
| Delivery | 0701 | | 3 | | | 17.82 | | | |
| **Total** |  | |  | | | **958.40** | | | |

1. Implant-supported porcelain-bonded crown

| Treatment | Position BEMA/GOÄ/GOZ/L | Number of treatments | Euro |
| --- | --- | --- | --- |
| Crown preparation | GOZ 2200 |  | 171.01 |
| Temporary crown | GOZ 2270 |  | 34.93 |
| Manipulation of abutments | GOZ 9050 | 2 | 80.98 |
| Individual impression | GOZ 5170 |  | 14.06 |
| Dental materials |  |  | 22.07 |
| *Laboratory* | | | |
| Situation model | 0010 | 3 | 17.22 |
| Individual tray | 0211 |  | 19.51 |
| Used resin | 0023 |  | 12.14 |
| Single-tooth dye | 0051 |  | 9.19 |
| Occludator | 0120 |  | 8.42 |
| Gingival mask | 0223 |  | 10.99 |
| Working with a supra-structure | 2971 |  | 25.77 |
| Working on abutment | 2973 |  | 47.47 |
| Crown core | 2122 |  | 70.26 |
| Porcelain coverage | 2612 |  | 99.83 |
| Non-precious metal alloy | 9700 |  | 11.68 |
| Delivery | 9330 | 6 | 25.98 |
| Impression post | 9237 |  | 56.54 |
| Laboratory implant | 9238 |  | 25.40 |
| Abutment and screw | 9239 |  | 84.82 |
| **Total** |  |  | **848.27** |

1. Dental materials (for laboratory work, dental material costs used in surgery are additionally charged by the dentist.
   1. Alginate impression (2.15)
   2. Siloxane impression (9.96)
   3. Bite registration material (9.96)
   4. per COT
      1. crown 2 alginate, 1 siloxane, 1 bite registration (22.07)
      2. post-core crown: 2 alginate, 2 siloxane, 2 bite registration (41.99)
