# Supplementary material for: Costs and Effectiveness of Treatment Alternatives for Proximal Caries Lesions
Source: PLoS One. 2014 Jan 27;9(1):e86992. doi: 10.1371/journal.pone.0086992 (PMC3903601; doi:10.1371/journal.pone.0086992)
Supplement: Text S1 — References for Supporting Information. (DOCX) [file pone.0086992.s006.docx]

References for the appendix

Al-Hiyasat AS, Barrieshi-Nusair KM, Al-Omari MA (2006). The radiographic outcomes of direct pulp-capping procedures performed by dental students. *The Journal of the American Dental Association* 137(12)*:*1699-1705.

Barthel CR, Rosenkranz B, Leuenberg A, Roulet J-F (2000). Pulp Capping of Carious Exposures: Treatment Outcome after 5 and 10 Years: A Retrospective Study. *Journal of Endodontics* 26(9)*:*525-528.

Burke FJT, Lucarotti PSK (2009). Ten-year outcome of crowns placed within the General Dental Services in England and Wales. *Journal of Dentistry* 37(1)*:*12-24.

Cho S-Y, Seo D-G, Lee S-J, Lee J, Lee S-J, Jung I-Y (2013). Prognostic Factors for Clinical Outcomes According to Time after Direct Pulp Capping. *Journal of Endodontics* 39(3)*:*327-331.

Dammaschke T, Leidinger J, Schäfer E (2010). Long-term evaluation of direct pulp capping—treatment outcomes over an average period of 6.1 years. *Clinical Oral Investigations* 14(5)*:*559-567.

Ferrari M, Vichi A, Fadda GM, Cagidiaco MC, Tay FR, Breschi L *et al.* (2012). A Randomized Controlled Trial of Endodontically Treated and Restored Premolars. *Journal of Dental Research* 91(7 suppl)*:*S72-S78.

Fitzgerald M, Heys RJ (1991). A clinical and histological evaluation of conservative pulpal therapy in human teeth. *Operative Dentistry* 16(3)*:*101-172.

GKV-Spitzenverband (2013). Official Statistics January [Amtliche Statistik KM1].

Higgins JPT, Green S, editors (2011). Cochrane Handbook for Systematic Reviews of Interventions. Version 5.10 (updated March 2011): The Cochrane Collaboration.

Lumley PJ, Lucarotti PSK, Burke FJT (2008). Ten-year outcome of root fillings in the General Dental Services in England and Wales. *International Endodontic Journal* 41(7)*:*577-585.

Martignon S, Ekstrand KR, Gomez J, Lara JS, Cortes A (2012). Infiltrating/Sealing Proximal Caries Lesions: A 3-year Randomized Clinical Trial. *Journal of Dental Research* 91(3)*:*288-292.

Matsuo T, Nakanishi T, Shimizu H, Ebisu S (1996). A clinical study of direct pulp capping applied to carious-exposed pulps. *Journal of Endodontics* 22(10)*:*551-556.

Mejàre I, Stenlund H, Zelezny-Holmlund C (2004). Caries Incidence and Lesion Progression from Adolescence to Young Adulthood: A Prospective 15-Year Cohort Study in Sweden. *Caries Research* 38(2)*:*130-141.

Ng YL, Mann V, Gulabivala K (2008). Outcome of secondary root canal treatment: a systematic review of the literature. *International Endodontic Journal* 41(12)*:*1026-1046.

Opdam NJM, Bronkhorst EM, Loomans BAC, Huysmans MCDNJM (2010). 12-year Survival of Composite vs. Amalgam Restorations. *Journal of Dental Research* 89(10)*:*1063-1067.

Pallesen U, van Dijken JWV, Halken J, Hallonsten A-L, Höigaard R (2013). Longevity of posterior resin composite restorations in permanent teeth in Public Dental Health Service: A prospective 8 years follow up. *Journal of Dentistry* 41(4)*:*297-306.

Paris S, Hopfenmuller W, Meyer-Lueckel H (2010). Resin Infiltration of Caries Lesions. *Journal of Dental Research* 89(8)*:*823-826.

Shovelton DS, Friend LA, Kirk EE, Rowe AH (1971). The efficacy of pulp capping materials. A comparative trial. *Br Dent J* 130(9)*:*385-391.

Torabinejad M, Anderson P, Bader J, Brown LJ, Chen LH, Goodacre CJ *et al.* (2007). Outcomes of root canal treatment and restoration, implant-supported single crowns, fixed partial dentures, and extraction without replacement: A systematic review. *The Journal of prosthetic dentistry* 98(4)*:*285-311.

Torabinejad M, Corr R, Handysides R, Shabahang S (2009). Outcomes of Nonsurgical Retreatment and Endodontic Surgery: A Systematic Review. *Journal of Endodontics* 35(7)*:*930-937.

Willershausen B, Willershausen I, Ross A, Velikonja S, Kasaj A, Blettner M (2011). Retrospective study on direct pulp capping with calcium hydroxide. *Quintessence Int* 42(2)*:*165-171.
